# Altered expression of the immunoregulatory ligand-receptor pair CD200-CD200R1 in the brain of Parkinson’s disease patients

**DOI:** 10.1038/s41531-022-00290-2

**Published:** 2022-03-16

**Authors:** Neus Rabaneda-Lombarte, José Manuel Vidal-Taboada, Tony Valente, Mario Ezquerra, Rubén Fernández-Santiago, María José Martí, Yaroslau Compta, Josep Saura, Carme Solà

**Affiliations:** 1 Department of Cerebral Ischemia and Neurodegeneration, Institut d’Investigacions Biomèdiques de Barcelona-Consejo Superior de Investigaciones Científicas (CSIC), Institut d’Investigacions Biomèdiques August-Pi i Sunyer (IDIBAPS), Barcelona, Spain; 2 Biochemistry and Molecular Biology Unit, School of Medicine, University of Barcelona, IDIBAPS, Barcelona, Spain; 3 Peripheral Nervous System Research Group, Vall d’Hebron Research Institute (VHIR), Barcelona, Spain; 4 Parkinson’s Disease and Movement Disorders Unit, Service of Neurology, Institute of Clinical Neurosciences, Hospital Clínic of Barcelona, Barcelona, Spain; 5 Department of Clinical and Experimental Neurology, Laboratory of Parkinson disease and other Neurodegenerative Movement Disorders: Clinical and Experimental Research, IDIBAPS, University of Barcelona, Barcelona, Spain; 6 Centro de Investigación Biomédica en Red de Enfermedades Neurodegenerativas, CIBERNED, Barcelona, Spain; 7 Institute of Neurosciences, University of Barcelona, Barcelona, Spain

**Keywords:** Parkinson's disease, Parkinson's disease, Cellular neuroscience, Chronic inflammation

## Abstract

Neuroinflammation, in which activated microglia are involved, appears to contribute to the development of Parkinson’s disease (PD). However, the role of microglial activation and the mechanisms governing this process remain uncertain. We focused on one inhibitory mechanism involved in the control of microglial activation, the microglia inhibitory receptor CD200R1, and its ligand CD200, mainly expressed by neurons. The human CD200R1 gene encodes two membrane-associated and two soluble protein isoforms and the human CD200 gene encodes full-length proteins (CD200full) but also truncated (CD200tr) proteins which act as CD200R1 antagonists. Little is known about their expression in the human brain under pathological conditions. We used human peripheral blood monocytes and monocyte-derived microglia-like cells from control subjects to characterize the expression of the CD200R1 mRNA variants, which showed stimulus-specific responses. We provide evidence of increased CD200R1 (mRNA variants and protein isoforms) and CD200 expression (CD200tr mRNA) in brain tissue of PD patients, mainly in the hippocampus, as well as increased CD200 expression (CD200full and CD200tr mRNAs) in iPSCs-derived dopaminergic neurons generated from skin fibroblasts of PD patients. Our results suggest that CD200-CD200R1 signalling is altered in PD, which may affect the microglial function and constitute a potential target in therapeutic strategies for PD.

## Introduction

The contribution of glial cells, mainly activated microglia, to the etiology, and the progression of Parkinson’s disease (PD) has been repeatedly postulated^1–3^. The results of genetic and imaging studies suggest that microglial alterations occur in the brain of PD patients^4–6^, although the precise mechanisms by which they are involved in the development of neuronal damage remain to be elucidated. Preclinical studies show that inhibition of the inflammatory response associated with activated microglial cells is neuroprotective in experimental models of PD. Nevertheless, clinical studies using anti-inflammatory approaches in PD patients have failed to achieve positive results to date, suggesting that new targets or different treatment time windows need to be explored (revised in ^7^ and ^8^).

In homeostasis, several inhibitory mechanisms maintain the microglia in a surveillant phenotype in the central nervous system (CNS). However, the presence of chronic microglial activation in the brain of patients with PD suggests that these inhibitory mechanisms are impaired^9–14^. In the present study, we focused our attention on one of these mechanisms, the CD200-CD200R1 ligand-receptor pair, a potential therapeutic target for controlling inflammation in the human brain^15–17^. The CD200R1 immune inhibitory receptor is expressed by myeloid cells, and therefore in microglial cells in the CNS. In the CNS, CD200 is mainly expressed by neurons, although it is also expressed by astrocytes and oligodendrocytes in pathological conditions.

A decrease in the expression of CD200 and/or CD200R1 has been described in the brain of multiple sclerosis^18,19^ and Alzheimer’s disease patients^20^. In addition, monocyte-derived macrophages from PD patients show alterations in the regulation of CD200R1 in response to an inflammatory stimulus^21^. Recently, two potential risk polymorphisms for PD have been described in the promoter region of the CD200R1 gene, associated with reduced transcriptional activity of the promoter^22^. Changes in the expression of CD200 and/or CD200R1 have also been described in animal models of neurological disorders^23–26^. In these experimental models, inhibition of the CD200-CD200R1 system has resulted in a negative outcome^27–29^ and the stimulation of CD200R1 in a better outcome of the pathology^30,31^. In a recent study, we showed changes in the expression of CD200 and CD200R1 in an experimental mouse model of PD and the neuroprotective effect of a CD200R1 agonist^32^. Altogether, these results suggest that the CD200-CD200R1 ligand-receptor pair is a potential pharmacological target for the treatment of neurodegenerative processes.

While the murine *Cd200r1* gene encodes a single CD200R1 protein^33^, the human *CD200R1* gene can generate four mRNA variants through alternative splicing^34^. Variants 1 and 4 (long mRNA variants) encode protein isoforms 1 and 4, which are transmembrane proteins. Variants 2 and 3 (short mRNA variants) encode protein isoforms 2 and 3, which are soluble truncated proteins that lack the transmembrane and the cytoplasmic domains. Human CD200R1 isoform 4 is homologous to the murine CD200R1^34^. Regarding CD200, both the mouse and human *CD200* genes generate full-length (CD200full) and truncated (CD200tr) mRNA variants through alternative splicing^35,36^. CD200full protein, which is the most abundant form, interacts with CD200R1 and activates signal transduction pathways resulting in the inhibition of the pro-inflammatory response or the potentiation of the anti-inflammatory response in microglial cells^16^. However, the CD200tr protein lacks the N-terminal region and, although it interacts with CD200R1, it does not induce signal transduction, and is considered a physiologic antagonist of CD200-induced suppression^35^. Although different functions may be attributed to the membrane and soluble CD200R1 protein isoforms and to the full-length and the truncated CD200 isoforms, there are no studies regarding the possible functional relevance of each CD200 and CD200R1 mRNA variant or protein isoform. In addition, to our knowledge, little attention has been paid to the expression of the different CD200 and CD200R1 mRNA variants or protein isoforms in neurological disorders.

The aim of the present work was to study possible changes in the CD200-CD200R1 system in the human brain in the context of PD. To this end, we determined the expression of CD200 and CD200R1 in post-mortem samples of the substantia nigra, frontal cortex, and hippocampus of PD patients, identifying mRNA variants and protein isoforms. Furthermore, we investigated specific correlations with clinical and anatomopathological data. We first used human monocyte cultures obtained from peripheral blood and monocyte-derived microglia-like cell cultures to characterize the expression of CD200R1 mRNA variants in human myeloid cells. In addition, we also determined the expression of CD200full and CD200tr mRNAs in induced pluripotent stem cell (iPSC)-derived dopaminergic (DAn) and non-dopaminergic neurons (non-DAn) obtained from PD patients and their corresponding controls as an independent validation in a humanized PD neural system.

## Results

### Differential expression of CD200R1 mRNA variants in human monocytes and monocyte-derived microglia-like cells

Although CD200 is highly expressed in the brain, mainly by neurons, the level of CD200R1 expression, which is only expressed by myeloid cells, is very low in this organ. Microglia, the most abundant myeloid cells in the brain, only account for 10–15% of all brain cells. This fact and the existence of four CD200R1 mRNA variants resulting from alternative splicing and encoding protein isoforms with potentially different functions make the detection of the different CD200R1 mRNA variants and protein isoforms in brain tissue challenging. For this reason, we first set up and optimized the detection of the four CD200R1 mRNA variants in human myeloid cell cultures. To this end, we used primary human monocyte cell cultures obtained from peripheral blood. Then, we also studied the expression of CD200R1 mRNA variants in human microglia-like cells obtained by differentiation of the peripheral blood monocytes.

**Fig. 1:**
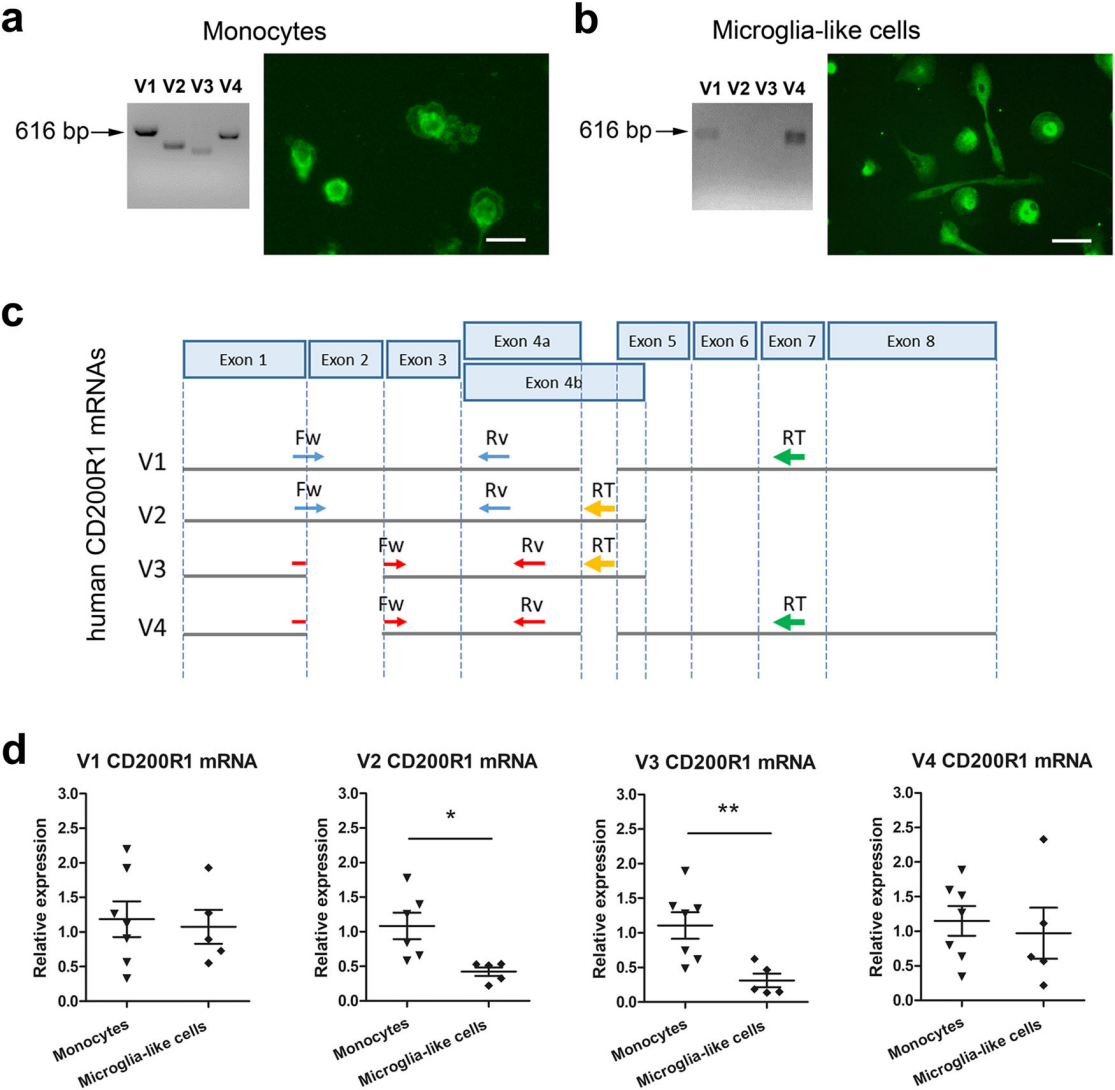
CD200R1 expression in human monocytes and microglia-like cells. Representative images of agarose gel electrophoresis illustrating conventional PCR products for CD200R1 mRNA variants after random primer retrotranscription (V1, 616 bp; V2, 468 bp; V3, 417 bp; V4, 565 bp) and CD200R1 protein expression by immunofluorescence (protein isoforms 1 and 4), in human monocytes cultured for 24 h (**a**) and microglia-like cells cultured for 14 days (**b**). Scale bar: 50 µm. **c** Schematic diagram of the localization of the mRNA target sequences of the primers used for the quantification of the four human CD200R1 mRNA variants by qRTPCR using gene-specific retrotranscription. Exons are not depicted to scale and are represented as rectangles. The arrows indicate the relative positions of primers: primers for the gene-specific retrotranscription (RT) of i) V1 and V4 long CD200R1 mRNA variants (green arrows) or ii) V2 and V3 short CD200R1 mRNA variants (yellow arrows); forward (Fw) and reverse (Rv) primer pairs for qRT-PCR that generate short amplicons to amplify V1 (blue arrows) or V4 (red arrows) in i) and V2 (blue arrows) or V3 (red arrows) in ii). **d** Comparative mRNA expression of the four CD200R1 mRNA variants in monocytes and microglia-like cells by qRT-PCR after gene-specific retrotranscription. *GAPDH* and *RPS18* were used as reference genes. Individual points are represented and bars indicate the positions of the mean ± SEM of 7 independent experiments. *p < 0.05 and **p < 0.01; two-tailed unpaired Student’s *t*-test.

We first detected the four CD200R1 mRNA variants in monocyte cultures by random primer retrotranscription followed by conventional PCR (Fig. 1a). We also detected V1 and V4 but not V2 and V3 CD200R1 mRNAs in microglia-like cells (Fig. 1b). We then corroborated the presence of CD200R1 protein in monocyte (Fig. 1a) and microglia-like cell cultures (Fig. 1b) by immunofluorescence, although the antibodies commercially available detect only the long transmembrane protein isoforms. As these experimental approaches are not quantitative, we next determined the expression of each CD200R1 mRNA by qRT-PCR.

However, because of the overlapping sequences of the four human CD200R1 mRNA variants (Fig. 1c), optimal primers (75–200 bp amplicon size) cannot be designed to individually quantify them by qRT-PCR after random primer retrotranscription. To quantify the expression of each variant, we designed an alternative approach based on gene-specific primer retrotranscription, which consists of specific primers for the retrotranscription of a) V1 and V4 variants (long mRNA variants) or b) V2 and V3 variants (short mRNA variants) (Fig. 1c), and primer pairs for qRT-PCR that generate short amplicons (75–200 bp) to amplify V1 or V4 in a) and V2 or V3 in b) (Fig. 1c) (Table 1). We first validated this approach using primary human monocyte cell cultures and we then compared the expression of each variant in monocytes and microglia-like cells. While V1 and V4 were similarly expressed in both cell types, microglia-like cells showed lower expression of V2 and V3 than monocytes (Fig. 1d). Altogether, these results suggest that CD200R1 is differentially expressed in peripheral blood monocytes and microglia.

**Table 1:** Primers used for conventional PCR and qRT-PCR.

Target mRNA	Accession number	Forward primer (5’→3’)	Reverse primer (5’→3’)	Amplicon size
CD200R1 PRIMERS USED FOR CONVENTIONAL PCR				
V1 CD200R1	NM_138806.4	GGTGCTGCTCAACCAAACAA	CCTCCCAGTGGCATGTACTCT	616 bp
V2 CD200R1	NM_138939.3	GGTGCTGCTCAACCAAACAA	CCTCAATATATGATGCTCCT	468 bp
V3 CD200R1	NM_138940.3	TTAGTGGCCGCTTCAAGCAG	CCTCAATATATGATGCTCCT	417 bp
V4 CD200R1	NM_170780.3	TTAGTGGCCGCTTCAAGCAG	CCTCCCAGTGGCATGTACTCT	565 bp
Reference gene:				
RPS18	NM_022551.3	CCTGAAAAGTTCCAGCATATTTTGC	TTTATTAACAGACAAGGCCTACAGAC	470 bp
PRIMERS USED FOR qRT-PCR				
After gene-specific retrotranscription:				
V1 and V2 CD200R1	NM_138806.4 NM_138939.3	ATCTTCTTAGTGGCCGAAGC	GCACAGCATTTGTAGCCATC	193 bp
V3 and V4 CD200R1	NM_138940.3 NM_170780.3	CTTCTTAGTGGCCGCTTCAA	TAGGAGGGCAACAAAGCACA	137 bp
After non-specific gene retrotranscription:				
V1 and V4 CD200R1	NM_138806.4 NM_170780.3	GTTGTTGAAAGTCAATGGCTGC	CACTTTGTAATGCCTCAGATGCC	164 bp
V2 and V3 CD200R1	NM_138939.2 NM_138940.3	TTCAGATTCGTACCGTGGCC	CCTCAATATATGATGCTCCT	125 bp
CD200full	Chen et al.^36a^	CAGCCTGGTTTGGGTCATG	GCAGAGAGCATTTTAAGGAAGCA	113 bp
CD200tr	Chen et al.^36^^b^	GATGGAGAGGCTGTGCAAGTG	GCAGAGAGCATTTTAAGGAAGCA	79 bp
Reference genes:				
ACTB	NM_001101.5	AGAGCTACGAGCTGCCTGAC	AGCACTGTGTTGGCGTACAG	184 bp
GAPDH	NM_002046.7	GAAGGTGAAGGTCGGAGTCA	GTTAAAAGCAGCCCTGGTGA	67 bp
RPS18	NM_022551.3	GATGGGCGGCGGAAAAT	CTTGTACTGGCGTGGATTCTGC	174 bp

*ACTB* Actin beta, *CD200full* full-length CD200, *CD200tr* truncated CD200, *CD200R1* CD200 receptor 1, *GAPDH* glyceraldehyde-3-phosphate dehydrogenase, *RPS18* ribosomal protein S18, *V1-V4 CD200R1* CD200 receptor 1 mRNA splice variant 1–4.

^a^ The sequences correspond to the primers published by Chen et al.^36^. Recent sequence updates show that they recognize 8 CD200 mRNA variants: variant 1, NM_005944.7; variant 2, NM_001004196.4; variant 3, NM_00131826.2; variant 6, NM_001365851.2; variant 7, NM_001365852.1; variant 8, NM_001365853.1; variant 9, NM_001365854.1; variant 10, NM_001365855.1. However, CD200 mRNA variants 3 and 10 encode a single CD200tr protein isoform (the same as CD200 mRNA variant 5).

^b^ The sequences correspond to the primers published by Chen et al.^36^. Recent sequence updates show that they recognize 2 CD200 mRNA variants: variant 4, NM_001318828.2; variant 5, NM_001318830.2. They encode two CD200tr protein isoforms.

**Fig. 2:**
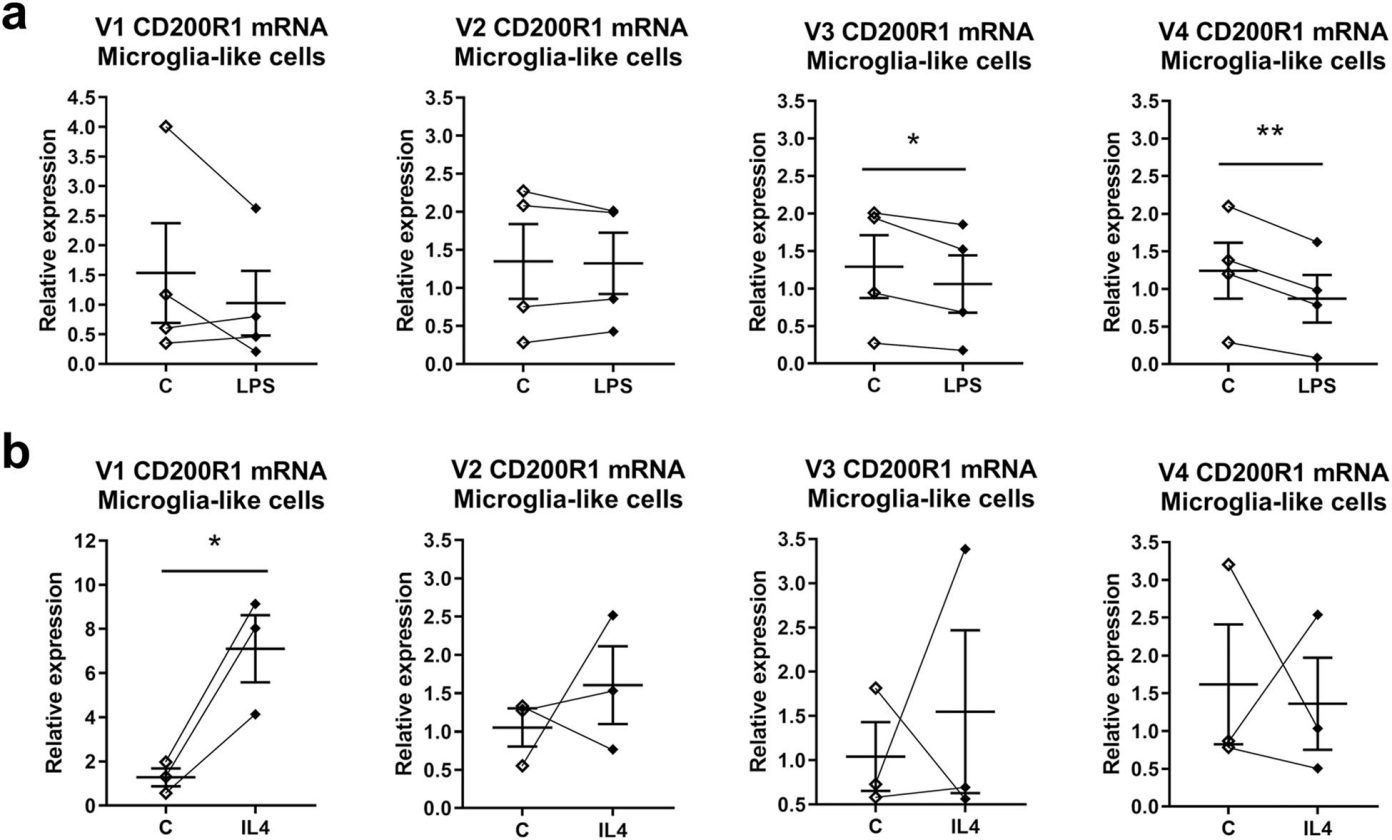
CD200R1 expression in human microglia-like cells treated with inflammatory stimuli. mRNA expression of the four CD200R1 mRNA variants in microglia-like cells treated for 24 h with LPS (100 ng/mL) (**a**) or IL4 (50 ng/mL) (**b**) by qRT-PCR after gene-specific retrotranscription. *GAPDH* and *RPS18* were used as reference genes. Individual points are represented and bars indicate the positions of the mean ± SEM of 3–4 independent experiments. *p < 0.05 and **p < 0.01; two-tailed paired Student’s *t*-test.

We also studied the expression of each CD200R1 mRNA variant in response to different stimuli, such as the pro-inflammatory stimulus LPS and the anti-inflammatory stimulus IL4. A significant decrease in the expression of V3 and V4 CD200R1 mRNA variants was observed in microglia-like cells treated with LPS for 24 h (Fig. 2a). On the contrary, a significant increase in V1 CD200R1 mRNA expression was detected 24 h after IL4 treatment (Fig. 2b).

### Changes in CD200R1 and CD200 mRNA expression in Parkinson’s disease

When analyzing each CD200R1 mRNA variant in post-mortem human brain tissue by conventional PCR, we detected V1 and V2 but not V3 and V4 mRNA variants. Then, using the same strategy as in monocytes and microglia-like cells to distinguish the four CD200R1 mRNA variants by qRT-PCR, we were able to detect the V1 mRNA variant, but not the other three CD200R1 mRNA variants. A low level of expression together with a dilution effect of microglial mRNAs in the human brain tissue mRNAs may be responsible for the lack of detection of all the CD200R1 mRNA variants.

**Fig. 3:**
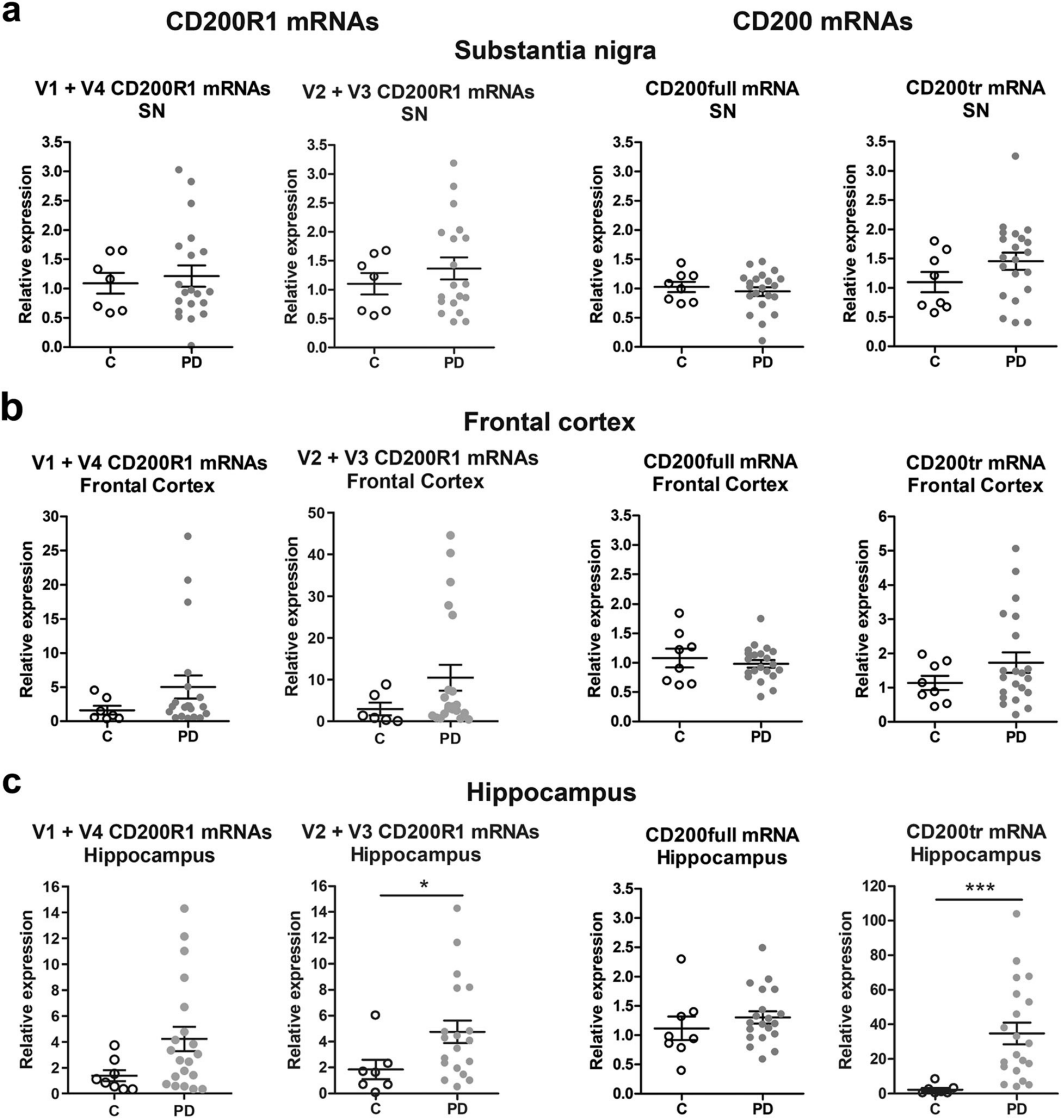
CD200R1 and CD200 mRNA expression in the post-mortem brain of Parkinson’s disease patients. Expression of long (V1 + V4) and short (V2 + V3) CD200R1 mRNA variants, and CD200full and CD200tr mRNAs in the substantia nigra (SN) (**a**), frontal cortex (**b**) and hippocampus (**c**) tissue homogenates from control individuals (C, *n* = 6–8) and Parkinson’s disease patients (PD, *n* = 19–21) by qRT-PCR. *GAPDH* and *RPS18* were used as reference genes. Data are expressed as fold change relative to C and are depicted as individual points with bars showing means ± SEM. *p < 0.05 and ***p < 0.001 vs. C; Mann-Whitney test. A maximum of one outlier or exceptionally two outliers were removed from each experimental group (Grubb’s test).

Given the singularity of CD200R1 expression in humans, where four mRNA variants are described instead of the one variant found in mice, we decided to study the expression of the two long variants together (V1+V4, which encode membrane-bound proteins) and the two short variants together (V2+V3, which encode soluble proteins). To this end, we used random primer retrotranscription followed by qRT-PCR with primers for long or short mRNA variants (Table 1). Using this methodology, we were able to detect the two types of variants in the agarose gel electrophoresis and also quantify them by qRT-PCR. Long and short CD200R1 mRNA variants were not differentially expressed in the substantia nigra and frontal cortex of PD subjects compared to age-matched controls, although a trend to increase was observed in the frontal cortex (Fig. 3a, b). However, short mRNA variants were significantly increased in the hippocampus of PD patients, where a trend to increase in long mRNA variants was also observed (Fig. 3c). As regards CD200, CD200full mRNA expression showed no differences between control individuals and PD patients in any of the three areas analyzed (Fig. 3a–c). On the contrary, CD200tr mRNA levels were strongly increased in the hippocampus from PD patients compared to control individuals (Fig. 3c).

### Changes in CD200R1 and CD200 protein expression in Parkinson’s disease

**Fig. 4:**
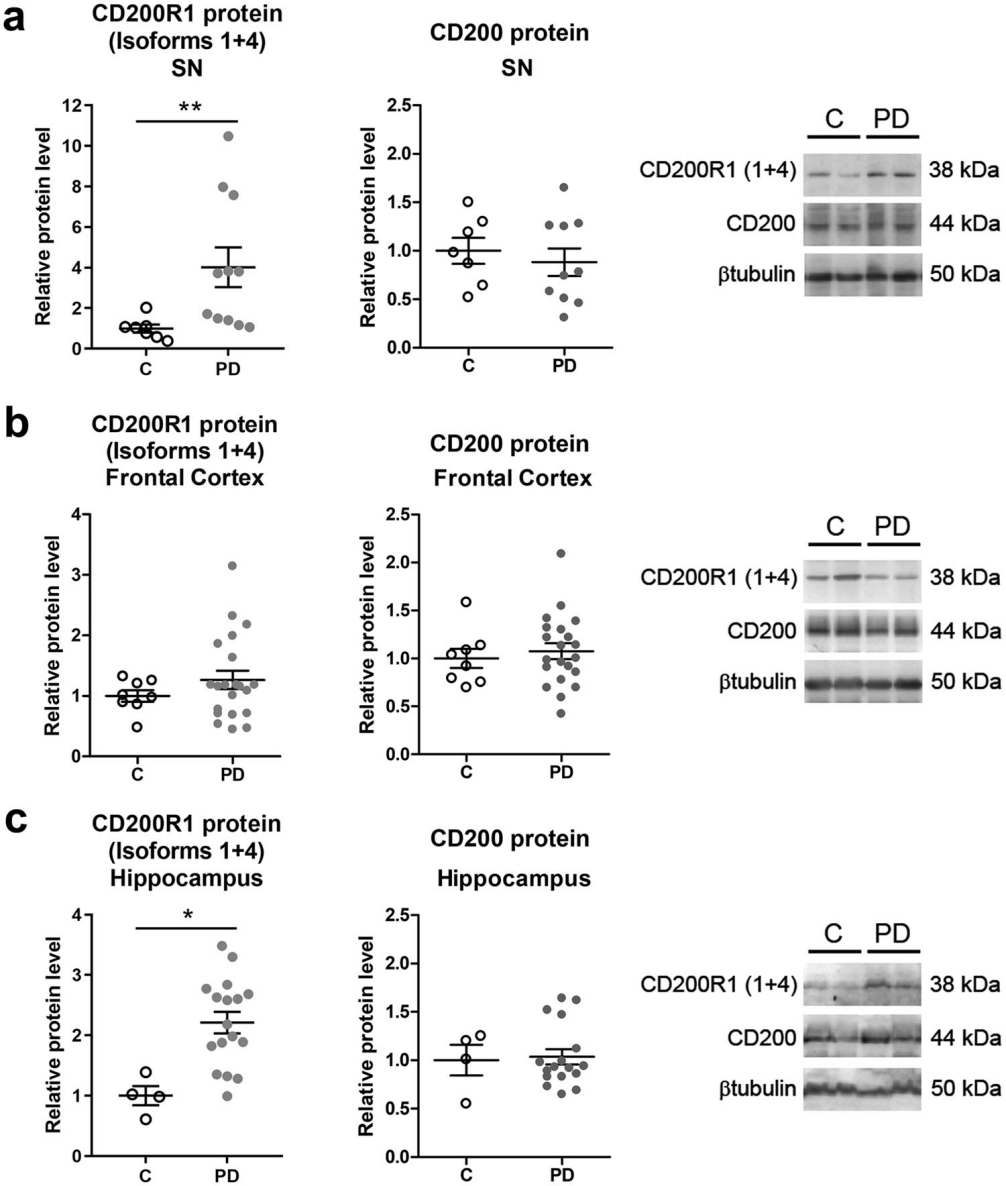
CD200R1 and CD200 protein levels in the post-mortem brain of Parkinson’s disease patients. Expression of CD200R1 protein isoforms 1 and 4 and CD200 proteins in the substantia nigra (SN) (**a**), frontal cortex (**b**), and hippocampus (**c**) tissue homogenates from control individuals (C, *n* = 4–8) and Parkinson’s disease patients (PD, *n* = 11–21) by western blot. Representative immunoblots are presented. Protein levels were normalized relative to βtubulin. Data are expressed as fold change relative to C and are depicted as individual points with bars showing means ± SEM. *p < 0.05 and **p < 0.01 vs. C; Mann-Whitney test.

We next studied the protein expression of CD200R1 and CD200 in the substantia nigra, frontal cortex, and hippocampus of PD patients and their corresponding controls. In the case of CD200R1, the antibodies commercially available detect CD200R1 long isoforms. In the case of CD200, the antibodies commercially available recognize CD200full and probably CD200tr as well. We observed higher levels of CD200R1 in the substantia nigra and hippocampus of PD patients than in age-matched controls, but no differences were detected in the frontal cortex (Fig. 4a–c). CD200 protein levels were not modified in the substantia nigra, frontal cortex, or hippocampus of PD patients when compared to controls (Fig. 4a–c).

### Correlation with clinical and anatomopathological data

To further study the expression of CD200 and CD200R1 in the brain of PD patients, we assessed whether the levels of expression correlated with specific clinical and anatomopathological data (Table 2).

**Table 2:** Clinical and anatomopathological data of cases.

Case No.	Gender	Age (years)	PMD (hours)	Anatomopathological examination	Clinical diagnosis	Age of onset (years)
C1	Male	78	6:00	Multi-infarct Leukoencephalopathy		
C2	Male	83	13:00	AgD I (mild)		
C3	Female	56	14:00	Brain metastasis oat cell lung cancer + NFT I-II		
C4	Female	86	4:00	Right vertebral thrombosis + cerebellar bulbar ictus + NFT III		
C5	Female	82	20:00	AgD III + NFT III + vascular encephalopathy		
C6	Female	90	12:20	Brainstem haemorrhage + SVD + ARP IIIB + TDP43 CA1		
C7	Male	78	6:00	iLBD Braak 1 + NFT I-II + SVD		
C8	Male	76	11:30	AgD I (minimal)		
PD1	Male	85	12:15	LP 5 + capillary CAA + glial tau	PD + dementia	Unknown
PD2	Male	82	7:15	LP 4-5 + AgD III + ARP IV B + microinfarcts	PD + dementia	Unknown
PD3	Male	76	17:10	LP 5 + ARP IVB	PD + dementia, DBS	51
PD4	Female	86	16:04	LP 6 + ARP IIIB	Parkinsonism	81
PD5	Female	82	13:10	LP 4 + ARP IB	PD	54
PD6	Male	79	11:30	LP 3 + NFT II	PD	Unknown
PD7	Male	50	16:30	LP 5 + hypoxic neuronal damage	PD, pallidum and subthalamic nucleus DBS	27
PD8	Male	81	5:00	LP 5 + AgD I	RBD + PD + dementia	71 (RBD) 74 (PD)
PD9	Female	87	7:00	LP 6 + ARP IVB	PD + dementia	67
PD10	Male	77	12:00	LP 4 + NFT II	RBD + PD + mild cognitive impairment	74
PD11	Female	78	18:00	LP 5 + glial tau + diffuse hypoxia	PD + Arnold Chiari I + IBM	56
PD12	Male	74	8:00	LP 5 + ARP IIIB + moderate CAA	PD	55
PD13	Male	87	15:15	LP 5 + ARP IIA	PD + dementia	71
PD14	Male	78	5:15	LP 5 + ARP IIB	PD + dementia	Unknown
PD15	Male	71	5:00	LP 4 + ARP IB	PD + dementia	Unknown
PD16	Male	74	14:03	Mild LP 5 + complex tauopathy	PD + dementia + hallucinations, Right pallidotomy	39
PD17	Male	81	7:20	LP 4-5 + ARP IIA + capillary CAA	PD	Unknown
PD18	Female	83	4:00	LP 4-5 + ARP IIA	PD + dementia + hallucinations	60 (right hand tremor) 76 (hallucinations + cognitive impairment)
PD19	Male	80	16:30	LP 4 + ARP II	PD + bilateral subthalamic nucleus DBS	40
PD20	Male	62	13:30	LP 5 + ARP IIA	PD + dementia	49-50 (PD) 58 (dementia)
PD21	Male	92	16:40	LP 4-5 + ARP IIIA + SVD	PD	76 (motor symptoms)

Cases identification: C1-C8, control cases; P1-P21, Parkinson’s disease cases.

*AgD* Argyrophilic grain disease, *ARP* Alzheimer’s disease-related pathology: classification of neurofibrillary tangle (NFT) pathology based on Braak staging^38^ (I-VI) and classification of neuritic plaques based on CERAD criteria (A-C)^39^; CAA: Cerebral amyloid angiopathy; CT: control; DBS: deep brain stimulation; IBM: Inclusion body myositis; LP: Lewy-pathology staging, studies based on the classification of Braak^37^ (1–6); iLBD: incidental Lewy body disease; PD: Parkinson’s disease; RBD: REM sleep behaviour disorder; PMD: post-mortem delay; SVD: small vessel disease; TDP43 CA1: TAR DNA binding protein 43 in hippocampal CA1 region.

**Fig. 5:**
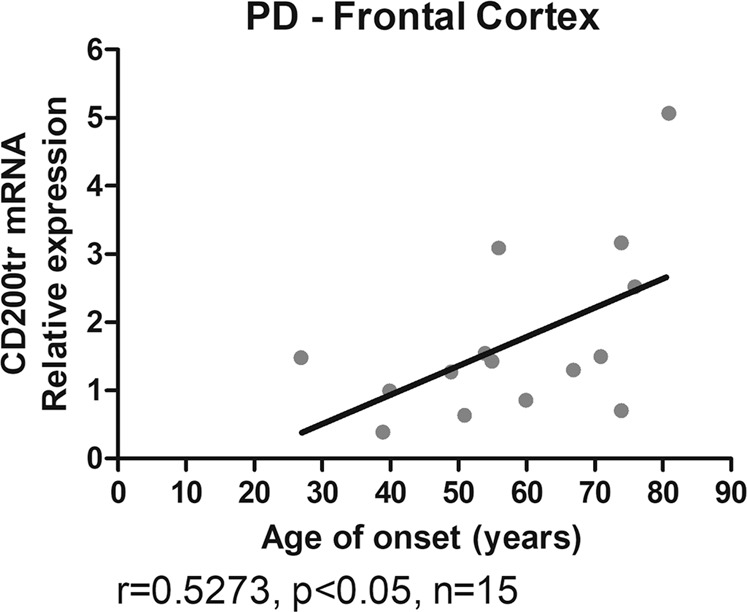
Significant correlation between CD200tr mRNA levels and age of onset in the frontal cortex in PD patients. Spearman’s correlation coefficient (*r*) and the corresponding p value are indicated.

In an analysis of our cohort according to clinical and demographic data, we considered parameters such as gender, age at death, age of onset of the disease, duration of the disease, and the presence of dementia. In PD patients, the age of onset was negatively correlated with the duration of the disease (*r* = −0.7502, *p* < 0.01, *n* = 15, Spearman correlation) while it was positively correlated with the age at death (*r* = 0.7359, *p* < 0.01, *n* = 15, Spearman correlation). In general, we observed no correlations between the level of expression of CD200R1, CD200full, and CD200tr and gender, age at death, age of PD onset, duration of the disease or presence of dementia. The only exception was that CD200tr mRNA levels in the frontal cortex of PD patients were positively correlated with age of onset (*r* = 0.5273, *p* < 0.05, *n* = 15, Spearman correlation) (Fig. 5). Multiple regression analysis showed that this effect was not driven by the age at death.

**Fig. 6:**
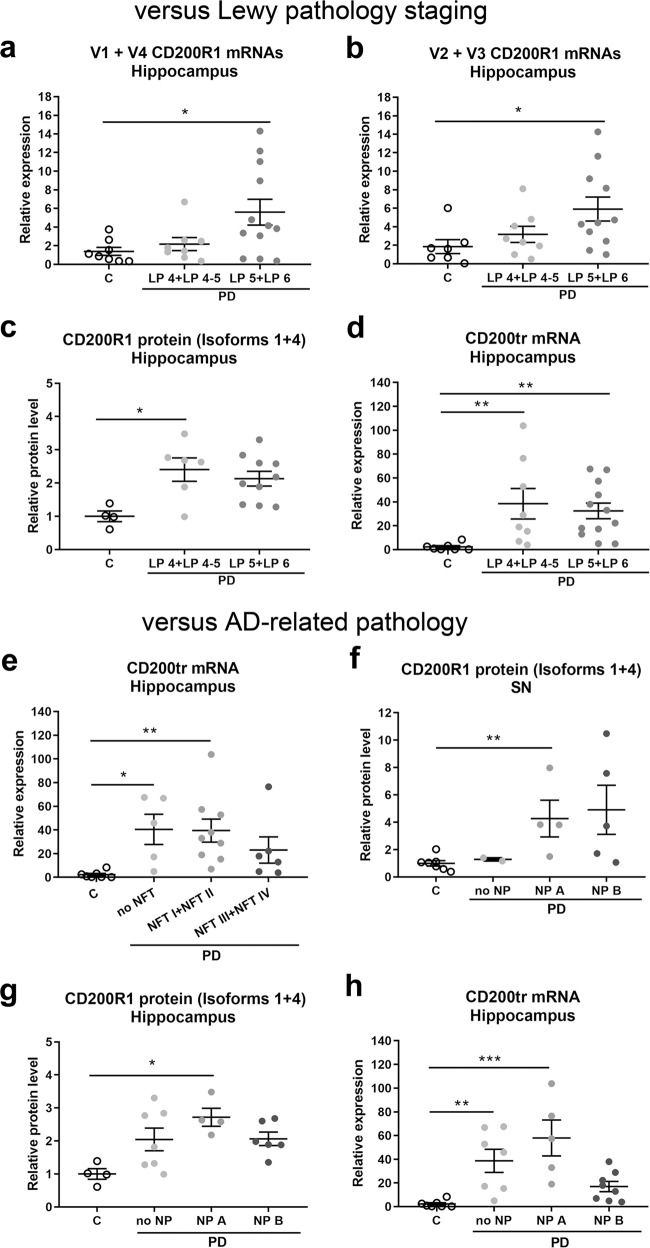
Significant associations between CD200R1 and CD200 expression levels in PD patients and anatomopathological data. **a**–**d** Expression levels versus the stage of Lewy body pathology. CD200R1 mRNAs (**a** and **b**), CD200R1 protein (**c**) and CD200tr mRNA expression (**d**) in the hippocampus of control (C, *n* = 4–8) and PD patients grouped according to the Lewy pathology staging (LP 4+LP 4–5, *n* = 6–8; LP 5–6, *n* = 10-12). LP 4+LP 4-5, Lewy pathology in medulla oblongata, pontine tegmentum, midbrain, basal prosencephalon, and mesocortex; LP 5-6, Lewy pathology in medulla oblongata, pontine tegmentum, midbrain, basal prosencephalon, mesocortex and neocortex. **e**–**h** CD200R1 and CD200 expression levels versus the stage of Alzheimer’s disease (AD)-related pathology. **e** CD200tr mRNA expression in the hippocampus of control individuals (C, *n* = 7) and PD patients grouped according to the topographical distribution pattern of AD-related neurofibrillary tangle pathology (NFT) (no NFT, *n* = 5; NFT I-II stages, *n* = 9; NFT III-IV stages, *n* = 6). No NFT, absence of NFT; NFT I and NFT II stages, transentorrinal areas; NFT III and NFT IV stages, transentorhinal and limbic areas. CD200R1 protein in substantia nigra (SN) (**f**), and CD200R1 protein (**g**) and CD200tr mRNA expression (**h**) in the hippocampus of control individuals (C, *n* = 4-7) and PD patients grouped according to the stage of AD-related neuritic plaque (NP) score (no NPs, *n* = 2–7; NP A, *n* = 4–5; NP B, *n* = 5–8). No NP, absence of NP; NP A, sparse neuritic plaques; NP B, moderate neuritic plaques. Data are expressed as fold change relative to C and are depicted as individual points with bars showing means ± SEM. *p < 0.05, **p < 0.01, and ***p < 0.001 vs. C; Kruskal-Wallis test and Dunn’s post hoc test.

We then analyzed whether the levels of CD200R1 and CD200 expression in PD patients were associated with anatomopathological alterations (Fig. 6). Thus, we first examined whether they were influenced by the stage of Lewy pathology (LP stage) (Fig. 6a–d). Lewy pathology staging was based on the classification of Braak et al.^37^: LP 1 stage, Lewy pathology in medulla oblongata; LP 2 stage, LP 1 plus pontine tegmentum; LP 3 stage, LP 2 plus midbrain; LP 4 stage, LP 3 plus basal prosencephalon and mesocortex; LP 5 and LP 6 stages, LP 4 plus neocortex. All the patients in our cohort presented LP 4-LP 6 stages, with the exception of one patient who presented LP 3 stage. In the substantia nigra and frontal cortex, no differences in CD200R1 and CD200 expression were found among the different LP stages. However, we observed higher levels of all CD200R1 mRNAs in the hippocampus of PD patients at advanced LP stages (LP 5 and LP 6) than at early LP stages (LP 4 and LP 4-5) (Fig. 6a, b); this effect was not observed at the protein level, which already showed a significant increase at LP 4 and LP 4-5 stage (Fig. 6c). In addition, CD200tr mRNA was similarly increased in the hippocampus of PD patients at all LP stages (Fig. 6d).

Next, we determined the influence of Alzheimer’s disease-related pathology, such as neurofibrillary tangles and neuritic plaques, on the results obtained (Fig. 6e–h). Firstly, the levels of CD200R1 and CD200 expression were analyzed versus the stages of neurofibrillary tangle pathology (NFT) defined by Braak et al. taking into account the topographical distribution pattern of the neurofibrillary lesions^38^: NFT I and NFT II stages, transentorhinal areas; NFT III and NFT IV stages, limbic areas; NFT V and NFT VI stages, isocortical areas. None of the patients in our cohort presented NFT V or NFT VI stages. In the substantia nigra and the hippocampus, the increased levels of CD200R1 protein observed in PD patients did not depend on the NFT stage. However, the increased CD200tr mRNA levels detected in the hippocampus of PD patients were higher in patients without neurofibrillary pathology or with NFT I + NFT II stage than with higher NFT stages (Fig. 6e). No relation between CD200 or CD200R1 levels of expression and stage of neurofibrillary degeneration in the frontal cortex was detected.

Finally, we analyzed the data taking into account the neuritic plaque score in the PD patients according to CERAD (Consortium to Establish a Registry for Alzheimer’s Disease) criteria^39^: sparse neuritic plaques (NP A), moderate neuritic plaques (NP B), and frequent neuritic plaques (NP C). None of the patients in our cohort presented NP C. We observed that in the substantia nigra and the hippocampus of PD patients, the highest increases in CD200R1 protein were detected in cases with NPA (Fig. 6f and g). Similarly, the highest increases in CD200tr mRNA in the hippocampus were detected in cases without Alzheimer’s disease-related pathology or with NP A (Fig. 6h).

### CD200 expression in iPSCs from controls and PD patients

**Fig. 7:**
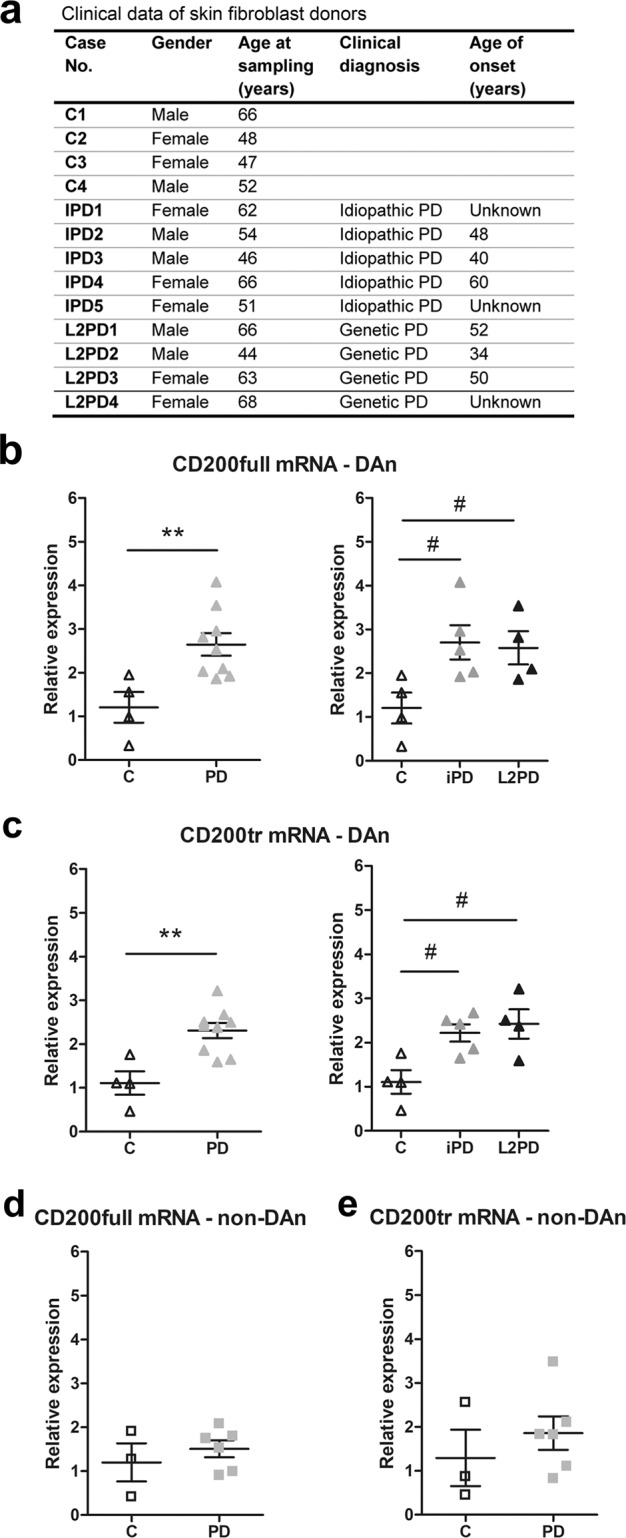
CD200 expression in iPSCs-derived neurons of PD patients and control subjects. **a** Clinical data of skin fibroblast donors. Cases identification: C1-C4, control cases; iPD1-iPD4, idiopathic Parkinson’s disease cases; L2PD1-L2PD4, genetic Parkinson’s disease cases carrying G2019S mutation in the LRRK2 gene. Expression of CD200full (**b** and **d**) and CD200tr mRNA (**c** and **e**) in iPSC-derived dopaminergic neurons (DAn) (**b, c**) and iPSC-derived neural cultures not-enriched in DAn (iPSC-derived non-DAn) (**d, e**) from PD patients (PD) and controls (C) by qRT-PCR. *GAPDH* and *ACTB* were used as reference genes. Individual points are represented and bars indicate positions of mean ± SEM of 3-6 cases. **p < 0.01 vs. C, two-tailed unpaired Student’s *t*-test; #*p* < 0.05 vs C, one-way ANOVA and Newman-Keuls post hoc test.

Finally, we determined the expression of CD200full and CD200tr mRNAs in iPSC-derived DAn generated from skin fibroblasts from PD patients and controls. This experimental approach is an interesting tool used to estimate the gene expression of specific neural cell types not necessarily representing end-stage disease nor being influenced by post-mortem factors such as post-mortem delay. We considered both patients bearing mutations in leucine-rich repeat kinase 2 (LRRK2) and sporadic idiopathic PD patients (Fig. 7a). We observed a significant increase in CD200full mRNA expression in DAn from PD patients, which was linked to both LRRK2-associated and sporadic idiopathic PD (Fig. 7b). CD200tr mRNA expression was also significantly increased in iPSC-derived DAn of all PD patients, an effect that was also significant when LRRK2-associated and sporadic idiopathic PD samples were analysed separately in independent comparisons (Fig. 7c). In contrast, CD200full and CD200tr mRNA expression were not significantly modified in iPSC-derived cultures not-enriched-in-DAn from the same PD patients relative to controls (Fig. 7d, e). These results show that the increased CD200 mRNA levels observed in iPSCs-derived neurons from PD patients are specifically associated with DAn.

## Discussion

The aim of the present study was to investigate possible alterations in the expression of the microglial inhibitory receptor CD200R1 and its ligand CD200 in the brain of PD patients. The existence of different mRNA variants encoding these proteins was taken into account, given the potential different functional significance of the resulting proteins. We used human monocyte and microglia-like cell cultures to optimize the protocol to quantify the four human CD200R1 mRNA variants by qRT-PCR. In the brain of PD patients, we detected an increase in the expression of CD200R1 and CD200tr when compared to controls. Among the three brain regions analyzed (substantia nigra, frontal cortex, and hippocampus), the hippocampus presented the most marked changes in expression in PD patients. Finally, we detected an increase in the expression of CD200full and CD200tr in iPSC-derived cultures of DAn generated from skin fibroblasts of PD patients when compared to controls.

The CD200-CD200R1 ligand-receptor pair plays an inhibitory role in the control of microglial activation in the CNS, contributing to the maintenance of microglial cells in a resting/surveillant condition under physiological conditions^15–17^. A decreased expression of CD200 and/or CD200R1 in the brain of Alzheimer’s disease^20^ and multiple sclerosis patients^18,19^ suggests that this inhibitory mechanism has been overloaded in these neurological disorders. Nevertheless, these studies do not discriminate between CD200full and CD200tr or among the different CD200R1 mRNA variants and the resulting protein isoforms. To our knowledge, this is the first time that the mRNA expression of all the components described in the human CD200-CD200R1 system (CD200full, CD200tr, and the CD200R1 variants) have been analyzed.

Quantification of the mRNA expression of each CD200R1 variant is not possible using random primer retrotranscription followed by qRT-PCR in optimal conditions (75–200 bp amplicon size) because of their overlapping sequences. Consequently, we designed a method based on gene-specific retrotranscription that we validated using human monocyte and microglia-like cell cultures. While monocytes clearly expressed the four CD200R1 mRNA variants, microglia-like cells presented V1 and V4 mRNA levels similar to monocytes but V2 and V3 mRNA levels much lower than in monocytes. In addition, the expression of CD200R1 mRNA variants in microglia-like cells was differentially affected by pro- and anti-inflammatory stimuli. These results indicate that myeloid cell types differ in their relative expression of the CD200R1 mRNA variants and suggest a stimulus-specific regulation of human CD200R1 mRNA variants. However, the function of each human CD200R1 protein isoform remains to be elucidated. Because of its amino acid sequence identity with murine CD200R1^34^, human protein isoform 4 most likely has similar functions to murine CD200R1.

In post-mortem brain tissue, we only detected the expression of V1 and V2 mRNAs by conventional PCR. It is necessary to take into account that the CD200R1 gene is only faintly expressed in microglial cells, which are present at a low percentage in the brain tissue. Consequently, the V3 and V4 variants may be present but the methodology may not be sensitive enough to detect them individually. Isolation of microglia from brain tissue to obtain pure microglia mRNA would be of relevance to study the expression of the CD200R1 mRNA variants in these cells. However, Vieites and collaborators described the presence of the four mRNA variants in different human tissues including the brain by conventional PCR^34^. These authors did not specify which region of the brain was analyzed. Differential expression of each CD200R1 mRNA variant in different brain regions could explain these discrepancies.

Given the singularity of CD200R1 expression in humans, where the CD200R1 gene encodes not only long transmembrane protein isoforms but also short soluble protein isoforms, and not ruling out the possible presence of the four CD200R1 mRNA variants in the brain tissue, we decided to study the possible differences between the mRNA expression of membrane vs. soluble mRNA variants. Thus, we quantified by qRT-PCR the long mRNA variants together (V1 + V4) and the short mRNA variants together (V2 + V3) in post-mortem brain from PD patients and the corresponding controls. The results obtained are summarized in Table 3. We detected increased expression in the short CD200R1 mRNAs (V2 + V3) encoding soluble forms of CD200R1 in the hippocampus of PD patients and a trend to increase in the frontal cortex. A trend to increase in CD200R1 mRNAs encoding the long transmembrane forms (V1 + V4) was also observed in the hippocampus and frontal cortex of PD patients; the increase was statistically significant in the hippocampus of PD patients with advanced stages of Lewy pathology (LP 5+LP 6). CD200R1 protein levels were increased in substantia nigra and hippocampus. An increase in the expression of the CD200R1 membrane forms may be interpreted as a potentiation of the CD200-CD200R1 system in the context of inflammation resolution and reparative response. On the contrary, although the physiological meaning of the CD200R1 soluble isoforms remains unknown, an increase might result in inhibition of the CD200-CD200R1 system by preventing the binding of cells expressing the ligand to cells carrying the membrane receptor, acting as decoy receptors.

**Table 3:** Summary of the alterations in CD200R1 and CD200 expression and the associated anatomopathology in the brain of Parkinson’s disease patients.

BRAIN AREA	CD200R1 EXPRESSION			CD200 EXPRESSION		
	Associated anatomopathology			Associated anatomopathology		
	mRNA variants		Protein (isoforms 1 + 4)	mRNA variants		Protein (isoforms full + truncated)
	V1+V4	V2+V3		full-length	truncated	
Substantia nigra	=	=	↑	=	=	=
			NP A			
Frontal cortex	(↑)	(↑)	=	=	=	=
Hippocampus	↑	↑	↑	=	↑	=
	LP 5+LP 6	LP 5+LP 6	LP 4+LP 4-5 NP A		LP 4+LP 4-5, LP 5+LP 6 no NFT, NFT I + NFT II no NP, NP A	

=, no changes; ↑, statistically significant increase; (↑), trend to increase.

In the substantia nigra and the hippocampus of Parkinson’s disease patients, some of the alterations in CD200R1 and CD200 expression detected were significantly associated with some anatomopathological markers (see Fig. 6): LP 4+LP 4-5, Lewy pathology in medulla oblongata, pontine tegmentum, midbrain, basal prosencephalon, and mesocortex; LP 5+LP 6, Lewy pathology in medulla oblongata, pontine tegmentum, midbrain, basal prosencephalon, mesocortex, and neocortex; no NFT, absence of neurofibrillary tangle pathology; NFT I and NFT II stages, neurofibrillary tangle pathology in transentorrinal areas; no NP, absence of neuritic plaques; NP A, sparse neuritic plaques.

V1 + V4 CD200R1 mRNAs, mRNA variants encoding long transmembrane CD200R1 protein isoforms; V2 + V3 CD200R1 mRNAs, mRNA variants encoding short soluble CD200R1 protein isoforms.

Few studies have considered the expression of CD200full and CD200tr separately, albeit only CD200full is thought to induce immunosuppression. Interestingly, Chen et al.^36^ showed that the susceptibility of different mouse strains to lung pathology after viral (MHV-1) infection is correlated with an increase in the CD200full/CD200tr ratio, because the balance shifts towards the immunosuppressive form. In addition, CD200tr expression in tumor cells stimulates tumor immunity and results in fewer metastases^40,41^. In the CNS, Matsumoto et al.^42^ described the presence of CD200 + macrophages in the ischemic regions of a rat stroke model; however, CD200tr was expressed at higher levels in the ischemic core and CD200full in the contralateral and peri-ischemic regions, suggesting that macrophages in the lesion core escape the suppression induced by CD200-CD200R1 interactions. In the experimental autoimmune encephalomyelitis model of multiple sclerosis, we detected changes in CD200 expression in several spinal cord regions, mainly a decrease in Cd200full mRNA expression and an increase in Cd200tr mRNA levels^24^. While Cd200full mRNA levels were negatively correlated with EAE clinical score, Cd200tr mRNA levels were positively correlated with EAE clinical score. In PD patients, we observed an increase in the expression of CD200tr mRNA in the hippocampus. Since it has been suggested that CD200tr may be a physiologic antagonist of CD200R1^35^, the interaction of CD200R1 membrane isoforms with CD200tr may interfere with CD200-CD200R1 signaling. On the contrary, the interaction of CD200R1 soluble isoforms with CD200full may interfere with CD200R1 signaling through cell-cell contacts, but their interaction with CD200tr may help to potentiate CD200-CD200R1 immunosuppressive function.

The primers used in the present study to distinguish CD200full and CD200tr mRNA expression in human samples by qRT-PCR were published by Chen et al. in 2010^36^. They designed the primers by considering the absence of *CD200* gene exon 2 (at present exon 3 in the NCBI database) in CD200tr mRNA as the distinctive trait differentiating between the two CD200 mRNA types. Recent sequence updates (NCBI database) reveal the existence of ten human CD200 transcript variants. The primers we used to detect CD200full mRNA recognize eight transcript variants that contain exon 3 (Table 1), while those used to detect CD200tr mRNA recognize two transcript variants without exon 3 (Table 1). Surprisingly, two of the transcripts targeted by CD200full primers (Table 1) encode a CD200tr protein isoform because exon 3 is not translated. Consequently, we were also quantifying these two CD200tr mRNA variants in our CD200full mRNA pool. However, this fact probably does not significantly affect the results obtained, as the contribution of these two CD200tr mRNAs to the total CD200full mRNA pool may be irrelevant. In fact, two CD200 mRNA variants are predominantly expressed in the human brain (GTEx Portal – Ensembl website), CD200 mRNA variant 1, which encodes a CD200full protein isoform, and CD200 mRNA variant 4, which encodes a CD200tr protein isoform. CD200 mRNA variant 1 is recognized by our CD200full mRNA primers and CD200 mRNA transcript variant 4 by our CD200tr mRNA primers. In fact, these two human CD200 mRNA variants are homologous to the two murine CD200 mRNA variants described to date (NCBI database).

In general, the changes we found in CD200R1 and CD200 expression in PD patients were more consistent in patients with advanced stages of Lewy pathology, a typical feature of PD (Table 3). In contrast, there was no clear association with characteristic features of Alzheimer’s disease, including neurofibrillary pathology or neuritic plaques, but PD patients with no or sparse neuritic plaques presented more changes in the CD200-CD200R1 system (Table 3). Walker and collaborators reported no differences in CD200 protein levels in the temporal and cingulate cortex of PD patients with or without dementia relative to controls^43^. At the protein level, we did not detect changes in CD200 expression in PD patients either, neither in the presence nor absence of dementia. However, when we distinguished the two CD200 mRNA variants, we detected a robust increase in CD200tr mRNA expression in the hippocampus of PD patients.

More studies are needed to clarify the functional role of each CD200R1 and CD200 isoform in the brain and the consequences of the changes in CD200R1 and CD200 expression observed in different brain areas in PD. To sum up, the results presented here show that the alterations in the CD200-CD200R1 system observed in PD, mainly increases in CD200R1 and CD200 expression, differ from those observed in Alzheimer’s disease^20^ and multiple sclerosis^18,19^, mainly decreases in CD200R1 and CD200 expression, suggesting that mechanisms associated with neuroinflammation differ among neurological disorders. The differences observed between the expression of long and short CD200R1 mRNA variants and between CD200full and CD200tr mRNAs emphasize the importance of taking into account the different variants separately when analyzing CD200R1 and CD200 expression. These differences may also be relevant in the development of drugs targeting the CD200/CD200R1 system, due to the opposite roles that the different CD200R1 and CD200 protein isoforms may have. The existence of all these CD200R1 and CD200 protein isoforms suggests a very complex mechanism of control of CD200-CD200R1 signaling and the resulting immunosuppression in the human brain.

The different patterns of expression of CD200R1 and CD200 observed in the substantia nigra, frontal cortex and hippocampus of PD patients may be related to the different degree of neuronal damage present in these brain areas. Massive cell death of the dopaminergic neurons in the substantia nigra pars compacta occurs in PD, which is responsible for the motor symptoms associated with the pathology. In the hippocampus of PD patients, where we detected the main changes in CD200R1 and CD200 expression, alpha-synuclein pathology and alterations in the cholinergic activity and in the expression of pro-inflammatory markers have been described^44–46^. However, no changes in the total volume or the number of neurons or glial cells have been detected in the hippocampus of PD patients^47^. These results suggest that neuronal dysfunction associated with microglial activation occurs in the hippocampus in the absence of neuronal death, which may be responsible for some nonmotor symptoms of PD such as cognitive deficits, memory decline, and visual hallucinations. Different microglial phenotypes may be associated with different degrees of neuronal damage. In this sense, Doorn et al.^48^ describe region-specific differences in the expression of different microglial phenotypes in substantia nigra and hippocampus of PD patients. In addition, Sawada et al.^46^ suggest that microglial activation may evolve from neuroprotective to neurotoxic with the progression of PD pathology.

We are looking at microglia as the brain cells expressing CD200R1 but monocytes and T cells also express this receptor. Different studies show that infiltrating monocytes^49^ as well as CD4 + ^45^ and CD8 + T cells^50^ can be found in the brain of PD patients. The potential role of peripheral immune cells and peripheral inflammation in the development of PD is a very interesting point to be taken into account in the context of the mechanisms involved in the development of PD, although this field is still underexplored. The fact that both peripheral immune cells and brain innate immune cells express CD200R1 and T cells also express CD200 provides additional complexity to the study of the CD200/CD200R1 system in the brain of PD patients. In addition, the presence of soluble forms that can enter or exit the brain adds further complexity to the subject.

Although post-mortem samples reflect an end-state of the disease, the results obtained using post-mortem brain tissue suggest the involvement of the mechanisms of control of the inflammatory response in PD development. Interestingly, an increase in CD200full and CD200tr mRNA expression was detected in iPSC-derived DAn generated from skin fibroblasts of PD patients, but not in the iPSC-derived cultures not-enriched-in-DAn. Although it is difficult to compare the observations in iPSC-derived neurons and in brain tissue, these results may indicate that changes in the CD200-CD200R1 ligand-receptor pair occurred specifically in DAn at modelled early stages of the disease although they were not observed at end stages of PD in post-mortem substantia nigra tissue samples, where 40–90% of DAn are dead. In a previous study, Fernandez-Santiago et al. described epigenomic and transcriptomic alterations in iPSC-derived DAn from PD patients, suggesting that possible developmental epigenetic defects are associated with an impaired DAn cellular identity in PD^51^. Moreover, in a recent paper, we also described an increase in α-synuclein mRNA expression in iPSC-derived DAn^52^. The results of the present study additionally suggest that some impairment in the mechanisms of neuron-glia communication may also occur in DA neurons in PD. The absence of effect in iPSC-derived cultures not-enriched-in-DAn in spite of the increase in CD200tr mRNA observed in the hippocampus needs further study. Apart from the difficulty of comparing the iPSC-derived neuronal cultures and the brain tissue mentioned above, differences between the main neuronal populations present in the iPSC-derived cultures not-enriched-in-DAn and in the hippocampus, or innervating the hippocampus, may account for the differences observed. In addition, different neuronal populations may be subsequently affected in PD following the course of the progression of the disorder (Braak staging).

In summary, changes in CD200 and CD200R1 expression occur during the development of PD, but the meaning of these changes, that is whether they are the result of cellular dysfunction or they participate in a compensatory response aimed at controlling the microglial inflammatory response, remains to be elucidated. An increase in CD200R1 expression, at least the membrane-associated forms, may be used as a therapeutic target for agonist compounds that could lead to the inhibition of the proinflammatory and neurotoxic potential of microglial activation. In fact, a neuroprotective effect of CD200R1 agonists has been described in experimental models of neurological disorders^31,32,53^. However, further studies are needed to characterize the potential of the mechanisms of control of microglial activation as possible therapeutic targets to control neuroinflammation and the derived neurotoxicity in PD. The longitudinal study of CD200 expression using iPSC-derived DAn and of CD200R1 expression in microglia-like cells derived from peripheral blood monocytes from PD patients represent promising tools that may help to understand the involvement of the CD200-CD200R1 system in the development of PD as well as its potential as a therapeutic target.

## Methods

### Ethical considerations

Monocyte cultures were prepared from peripheral blood cells of healthy adult volunteers. Peripheral blood samples were collected in collaboration with the Parkinson’s Disease Unit of the *Hospital Clínic de Barcelona*. Informed consent was obtained from blood donors, and the study was approved by the Ethics Committee of the *Hospital Clínic de Barcelona* (Ref. HCB/2021/0349).

Post-mortem human brain samples were supplied by the *Banc de Teixits Neurològics* (Biobanc, Hospital Clínic de Barcelona, IDIBAPS, Barcelona, Spain) (Ref. HCB/2014/1027) in accordance with the Helsinki Declaration, Convention of the Council of Europe on Human Rights and Biomedicine and Ethical Committees of the University of Barcelona and CSIC (Ref. 711/14).

### Human monocyte and microglia-like cell cultures

Sixty mL of peripheral blood from nine healthy adult volunteers (age range 23–66 years; five females and four males) were collected using 18 mg EDTA tubes (BD Biosciences, Madrid, Spain) and immediately processed. Peripheral blood mononuclear cells were isolated by Histopaque-1077 (Sigma-Aldrich, Madrid, Spain) density gradient centrifugation following the manufacturer’s protocol. Three 50 mL tubes were filled with 20 mL of Histopaque-1077 each and brought to room temperature. Twenty mL of blood were gently layered onto the Histopaque-1077, taking care not to mix the two liquids. The samples were centrifuged at 400 g for 30 min at room temperature. During centrifugation, erythrocytes aggregate and rapidly sediment, granulocytes become slightly hypertonic resulting in pelleting at the bottom of the tube, and lymphocytes and other mononuclear cells form a band at the interface between Histopaque-1077 and the plasma. After centrifugation, the opaque interface containing the mononuclear cells (approximately 4 mL) was carefully transferred from each 50 mL tube into a 15 mL tube and washed three times by adding 10 mL of culture medium and centrifuging at 250 g for 10 min. In the last wash, the three pellets were collected in a single 15 mL tube. The culture medium consisted of RPMI-1640 Glutamax (Invitrogen, Thermo Fisher Scientific, Madrid, Spain) supplemented with 10% heat-inactivated fetal bovine serum (Life Technologies, Thermo Fisher Scientific, Madrid, Spain), 100 U/mL penicillin—100 μg/mL streptomycin (Life Technologies, Thermo Fisher Scientific), and 0.25 μg/mL amphotericin B (Life Technologies, Thermo Fisher Scientific). Peripheral blood mononuclear cells were counted using a Neubauer chamber, plated onto 6-well plates (2 mL per well) or 48-well plates (200 µL per well) at a density of 4 ×10^5^ cells/mL and cultured at 37 °C in a 5% CO_2_ humidified atmosphere. The next day, culture medium and nonadherent cells were aspirated. The remaining adherent cells correspond to monocytes and were used at this time point.

Human microglia-like cell cultures were obtained from monocytes with a combination of the cytokines GM-CSF and IL-34 as previously reported^54^. Briefly, the monocytes, obtained as mentioned above, were cultured with RPMI-1640 Glutamax supplemented with 100 U/mL penicillin, 100 μg/mL streptomycin, 0.25 μg/mL amphotericin B, and a mixture of recombinant human GM-CSF (10 ng/mL; R&D Systems, Madrid, Spain) and recombinant human IL34 (100 ng/mL; Peprotech, Bionova Científica, Madrid, Spain). After 7 DIV, the medium containing cytokines was changed to medium without cytokines and the cell cultures were used at 14 DIV. This protocol has been shown to result in cells that are very similar to authentic microglia. Apart from expressing classical myeloid markers (PU.1 and Iba1) and microglial specific markers (TMEM119 and P2YR12), they show reduced expression of CD45, CD14, and CD200R when compared to human monocyte-derived macrophages and decreased CCR2 when compared to human monocytes and similar responses to IL-4 and dexamethasone than authentic microglia^54,55^.

Cells were treated with LPS from *Escherichia coli* (100 ng/mL; 026:B6; Sigma-Aldrich) or IL4 (50 ng/mL; recombinant mouse IL4 expressed in CHO cells, Creative BioMart, Shirley, NY, USA) and processed after 24 h. Control and treated cells were derived from the same donor. A stock solution of 1 mg/mL LPS in a serum-free culture medium was prepared and stored at −20 °C. A stock solution of 50 mg/mL IL4 in a mixture of milli-Q H2O: culture medium (1:1) was prepared and stored at −20 °C. A new aliquot was used in each experiment. The agents were added directly to the culture medium.

### Human brain tissue samples

Frozen tissue blocks containing substantia nigra, hippocampus, and frontal cortex were obtained from eight control subjects (age range 56–90 years, post-mortem delay range: 4–20 h) and twenty-one PD patients (age range 50–92 years, post-mortem delay range: 5–18 h) and stored at −80 °C until further processing (Table 2).

### iPSCs from control and PD patients

We used mature iPSC-derived DAn generated from skin fibroblasts from PD patients and healthy controls. Patient and cell line characterization of the samples used here are described in Fernandez-Santiago et al.^51^ and Sanchez-Danes et al.^56^. The reprogramming and differentiation protocols used are described in detail by Sanchez-Danes et al.^57^. Short demographic information on donors is given in Fig. 7a. We used samples from LRRK2-associated PD patients carrying the G2019S mutation (L2PD, *n* = 4) and sporadic idiopathic PD patients lacking a family history of PD and mutations in known PD genes (iPD, *n* = 5), as well as samples from healthy controls without a history of neurological disease (controls, *n* = 4). As a cellular control of iPSC-derived DAn, iPSC-derived non-DAn were generated as technical controls from a subset of representative PD patients (*n* = 6) and healthy subjects (*n* = 3) as previously described^51^. We analyzed RNA samples extracted from iPSC-derived DAn and iPSCs-derived non-DAn at 3 weeks of culture.

### RNA extraction, conventional PCR, and quantitative real-time PCR (qRT-PCR)

Isolation of RNA was performed in 1 DIV monocyte cultures or 14 DIV microglia-like cell cultures. Four-six wells from 6-well plates (monocytes) or twelve wells from 48-well plates (microglia-like cells) were used. Total mRNA was isolated using the PureLink RNA micro kit (Invitrogen, Thermo Fisher Scientific) according to the manufacturer’s instructions. In the case of frozen tissue samples, total RNA was extracted using the Trizol method (Tri®Reagent, Sigma-Aldrich) according to the manufacturer’s instructions. Total RNA concentration was measured on a Nanodrop 1000 (Thermo Fischer Scientific). The resulting RNA was stored at −80 °C until further use.

Two to five hundred ng of RNA (cultured cells) or one μg of RNA (tissue samples) were reverse transcribed with random and oligo(dT) primers using a qScriptTM cDNA Synthesis Kit (Quanta Biosciences) or with gene-specific primers (Fig. 1c) using a qScriptTM Flex cDNA Synthesis Kit (Quanta Biosciences) according to the manufacturer’s instructions. The primers (Integrated DNA Technology, IDT, Skokie, IL, USA) used for gene-specific retrotranscription were: 5′-AACTGGAGTAGATTCTG-3′ (V1 and V4 CD200R1), 5′-CTGGTGATGTGAAATAC-3′ (V2 and V3 CD200R1), 5′-CATACTTCTCATGGTTC-3′ (GAPDH) and 5′-CACGAAGGCCCCAGAA-3′(RSP18). The reverse transcription was performed using a thermal cycler under the following protocol: 25 °C for 5 min, 42 °C for 30 min and 85 °C for 5 min (in the case of random and oligo(dT) primers) or 65 °C for 5 min, 42 °C for 45 min, and 85 °C for 5 minutes (in the case of gene-specific primers). In the case of iPSCs, total RNA extraction and reverse transcription were performed as described elsewhere^51^. The resulting cDNA was stored at −20 °C until further use.

The cDNA was diluted 1/10 to perform conventional PCR. Specific primers (Integrated DNA Technology) for each mRNA were used (Table 1). Conventional PCR was carried out using 2x PCRBIO Ultra Mix (PCR Biosystems, Cultek, Madrid, Spain) in 20 µL of final volume according to the manufacturer’s instructions in a thermal cycler under the following protocol: 95 °C for 2 min followed by 37 cycles consisting of 95 °C for 20 s; 60 °C for 35 s; and 72 °C for 30 s. The amplified DNA was loaded onto an agarose gel, together with a DNA ladder (Thermo Scientific, Thermo Fisher Scientific). For DNA detection, Midori green nucleic acid staining solution was used (Nippon Genetics Europe, Cultek, Madrid, Spain) and images were obtained using an UV Transilluminator (Gel Doc System, Bio-Rad Laboratories, Inc., Madrid, Spain).

The cDNA was diluted 1/10 (cultured cells) or 1/30 (tissue samples) and 3 ng (cultured cells) or 5 ng (tissue samples) of cDNA were used to perform qRT-PCR with SYBR Green Mix (PCR Biosystems) in 15 µL of final volume, using a C1000 Thermal Cycler CFX96 apparatus (Bio-Rad Laboratories, Inc.). Samples were run at 95 °C for 2 minutes to activate the polymerase followed by 40 cycles consisting of denaturation at 95 °C for 15 s, annealing at 60 °C for 30 s and extension at 72 °C for 15 s. The primers used (Integrated DNA Technology) are shown in Table 1 and the localization of the mRNA target sequences is represented in Fig. 1c. Relative gene expression values were calculated using the 2^−ΔΔCt^ method^58^. *GAPDH*, *RPS18*, and *ACTB* were used as reference genes.

### Immunocytochemistry

Cultured cells were fixed with 4% paraformaldehyde in 0.1 M phosphate buffer (pH 7.4) for 20 min at room temperature. Cells were then incubated overnight at 4 °C with goat polyclonal anti-CD200R1 primary antibody (AF2554, 1:100; R&D). Once they had been rinsed in PBS, cells were incubated for 1 h at room temperature with donkey anti-goat ALEXA 488 secondary antibody (A11055 488, 1:1000; Invitrogen). Antibodies were diluted in 0.3% Triton X-100 in PBS containing 1% BSA and 10% normal donkey serum. Microscopy images were obtained with an Olympus IX70 microscope (Olympus, Okoya, Japan) and a digital camera (CC-12, Olympus Soft Imaging Solutions GmbH, Hamburg, Germany).

### Total protein extraction and western blot

Total protein extracts were obtained from human tissue samples. Pieces of frozen tissue were ground in a mortar cooled with liquid nitrogen. Aliquots of approximately 100 mg were used for protein extraction. Total tissue protein was isolated in sodium dodecyl sulfate (SDS) buffer (0.125 M Tris-HCl pH 6.8, 2% SDS, 10% glycerol, 0.001% bromophenol blue, and 5% 2-mercaptoethanol) (1 mL/100 mg tissue)^59,60^. After vortexing and incubation for 10 min at 70 °C, samples were centrifuged at 16,100 g for 10 min at room temperature and the supernatant was collected. Samples were kept at −20 °C until further use. Protein concentration was determined using the Bio-Rad Protein Assay Kit (Bio-Rad Laboratories, Inc.), based on the Bradford assay.

For each sample, 30 μg of protein were diluted in loading buffer (120 mM Tris HCl pH 6.8, 10% glycerol, 3% SDS, 20 mM dithiothreitol, 0.4% bromophenol blue) and denatured at 100 °C for 5 minutes. Following a standard protocol^61^, the protein solutions were resolved by SDS-polyacrylamide gel electrophoresis (PAGE) on 12% polyacrylamide gels, together with a molecular weight marker (PageRuler™ Plus Prestained Protein Ladder, Thermo Scientific, Thermo Fisher Scientific). Proteins were transferred to polyvinylidene difluoride membranes (Merck Millipore, Madrid, Spain) by the traditional wet transfer method for 120 minutes at 60 V in transfer buffer (25 mM Tris pH 8.3, 192 mM glycine, 0.02% SDS, 20% methanol).

Membranes were blocked for non-specific interactions with 5% non-fat dry milk in Tris-Buffered Saline (TBS)-Tween 20 (20 mM Tris pH 7.5, 150 mM NaCl, 0.05% Tween-20) for 1 h at room temperature and incubated overnight at 4 °C with goat polyclonal anti-CD200R1 (sc-14392 M-21, 1:250; Santa Cruz Biotechnology Inc., Dallas, Texas USA) or anti-CD200 (AF3355, 1:500; R&D) or mouse monoclonal anti-βtubulin (T4026, 1:50,000; Sigma-Aldrich) primary antibodies. Then, the membranes were incubated with the corresponding horseradish peroxidase (HRP)-conjugated rabbit antigoat (P0449, 1:2000; Dako) or goat antimouse (170–6516, 1:5000; Bio-Rad Laboratories, Inc.) secondary antibodies for 1 h at room temperature, followed by repeated washing with TBS-Tween 20. The signal was developed using the western blotting detection kit WesternBrith™ Sirius HRP substrate (Advansta, Ecogen, Barcelona, Spain). All blots or gels derive from the same experiment and were processed in parallel. Images were obtained using a VersaDoc System (Bio-Rad Laboratories, Inc.). Data were expressed as the ratio between the band intensity of the protein of interest and the loading control protein (βtubulin).

### Data presentation and statistical analysis

The results are presented in scatter dot plots and the mean ± standard error of the mean (SEM). All data were statistically analyzed with GraphPad Prism 8.0.1 software. Normality of data was determined by Shapiro-Wilk and D’Agostino and Pearson omnibus normality tests. When comparing two groups, statistical analyses were performed using two-tailed unpaired or paired Student’s *t*-test or the Mann-Whitney *U* test. When comparing more than two groups, statistical analyses were performed using one-way analysis of variance (ANOVA) followed by the Newman-Keuls post hoc test or the Kruskal-Wallis nonparametric one-way ANOVA followed by the Dunn’s post hoc test. Outliers were determined by the Grubb’s test. The Spearman correlation coefficient (*r*) was calculated to measure the linear correlation between the clinical PD data and mRNA or protein expression levels. Values of *p* < 0.05 were considered statistically significant.

### Reporting Summary

Further information on research design is available in the Nature Research Reporting Summary linked to this article.

## Supplementary information

Reporting Summary Checklist

## Data Availability

All data generated or analysed during this study are available from the corresponding author upon reasonable request.
