# Supplementary material for: Altered expression of the immunoregulatory ligand-receptor pair CD200-CD200R1 in the brain of Parkinson’s disease patients
Source: NPJ Parkinsons Dis. 2022 Mar 16;8:27. doi: 10.1038/s41531-022-00290-2 (PMC8927151; doi:10.1038/s41531-022-00290-2)
Supplement: Supplementary file 1 — Reporting Summary Checklist [file 41531_2022_290_MOESM1_ESM.pdf]

nature portfolio

Corresponding author(s): Carme Solà

Last updated by author(s): Nov 8, 2021

Reporting Summary

Nature Portfolio wishes to improve the reproducibility of the work that we publish. This form provides structure for consistency and transparency in reporting. For further information on Nature Portfolio policies, see our Editorial Policies and the Editorial Policy Checklist.

Statistics

For all statistical analyses, confirm that the following items are present in the figure legend, table legend, main text, or Methods section.

n/a	Confirmed	
☐	☒	The exact sample size (*n*) for each experimental group/condition, given as a discrete number and unit of measurement
☐	☒	A statement on whether measurements were taken from distinct samples or whether the same sample was measured repeatedly
☐	☒	The statistical test(s) used AND whether they are one- or two-sided<br>*Only common tests should be described solely by name; describe more complex techniques in the Methods section.*
☐	☒	A description of all covariates tested
☐	☒	A description of any assumptions or corrections, such as tests of normality and adjustment for multiple comparisons
☐	☒	A full description of the statistical parameters including central tendency (e.g. means) or other basic estimates (e.g. regression coefficient) AND variation (e.g. standard deviation) or associated estimates of uncertainty (e.g. confidence intervals)
☒	☐	For null hypothesis testing, the test statistic (e.g. *F*, *t*, *r*) with confidence intervals, effect sizes, degrees of freedom and *P* value noted<br>*Give P values as exact values whenever suitable.*
☒	☐	For Bayesian analysis, information on the choice of priors and Markov chain Monte Carlo settings
☒	☐	For hierarchical and complex designs, identification of the appropriate level for tests and full reporting of outcomes
☒	☐	Estimates of effect sizes (e.g. Cohen's *d*, Pearson's *r*), indicating how they were calculated

Our web collection on statistics for biologists contains articles on many of the points above.

Software and code

Policy information about availability of computer code

Data collection	Gel images: UV Transilluminator and Gel Doc System (Bio-Rad laboratories, Inc.).<br>Immunofluorescence images: Olympus IX70 microscope (Olympus) and CC-12 digital camara (Olympus).<br>Quantitative real-time PCR data: C1000 Thermal Cycler CFX96 and CFX Manager Software Version 3.1 (Bio-Rad Laboratories, Inc.).<br>Western blot data: Versadoc System and Quantity One 1-D Analysis Software Version 4.6.9 (Bio-Rad Laboratories, Inc.).
Data analysis	Microsoft Excel; GraphPad Prism Version 8.0.1.

For manuscripts utilizing custom algorithms or software that are central to the research but not yet described in published literature, software must be made available to editors and reviewers. We strongly encourage code deposition in a community repository (e.g. GitHub). See the Nature Portfolio guidelines for submitting code & software for further information.

Data

Policy information about availability of data

All manuscripts must include a data availability statement. This statement should provide the following information, where applicable:
- Accession codes, unique identifiers, or web links for publicly available datasets
- A description of any restrictions on data availability
- For clinical datasets or third party data, please ensure that the statement adheres to our policy

All data generated or analysed during this study are available from the corresponding author upon reasonable request.

Field-specific reporting

Please select the one below that is the best fit for your research. If you are not sure, read the appropriate sections before making your selection.

☒ Life sciences ☐ Behavioural & social sciences ☐ Ecological, evolutionary & environmental sciences

For a reference copy of the document with all sections, see nature.com/documents/nr-reporting-summary-flat.pdf

Life sciences study design

All studies must disclose on these points even when the disclosure is negative.

Sample size	No sample size calculation was performed. Primary cultures: 3-7 cultures; human tissue samples: a total of n=8 control subjects and n=21 Parkinson's disease patients; iPSCs derived neuronal culture samples: a total of n=4 samples from control cultures and n=9 samples from Parkinson's disease patient cultures. Samples sizes allowed us to detect stadistically significant differences among the experimental groups.
Data exclusions	Human samples: in the case of qRTPCR analyses of human brain samples, some data showing very extreme values were excluded as outliers (Outlier calculator GraphPad).
Replication	Primary cell cultures (monocyte and microglia-like cell cultures): a pool of cell culture wells was considered for each experimental condition. Experiments were repeated at least three times.<br>Human tissue samples: samples from 8 control subjects and twenty-one Parkinson's disease patients were used; DNA sample triplicates were considered in qRTPCR experiments and relative fold increases/decreases in gene expression were calculated versus control samples and reference genes; data were expressed as the ratio between the band intensity of the protein of interest and the loading control protein in western blot experiments.<br>iPSCs derived neuronal cultures experiments: DNA samples from a total of n=4 control cultures and n=9 Parkinson's disease patient cultures were used.
Randomization	Primary cultures: cell culture wells were divided into control or treated. Human tissue samples and DNA samples from iPSCs derived neuronal cultures: samples were obtained from control subjects and from Parkinson's disease patients.
Blinding	The samples were carefully numbered and labelled when collected and the nomenclature was maintained throughtout the processement of the samples. Blinding was not relevant to our study because the results and the conclusions were obtained from quantificable measures, not affected by subjectivity.

Reporting for specific materials, systems and methods

We require information from authors about some types of materials, experimental systems and methods used in many studies. Here, indicate whether each material, system or method listed is relevant to your study. If you are not sure if a list item applies to your research, read the appropriate section before selecting a response.

Materials & experimental systems

n/a	Involved in the study	
☐	☒	Antibodies
☒	☐	Eukaryotic cell lines
☒	☐	Palaeontology and archaeology
☒	☐	Animals and other organisms
☐	☒	Human research participants
☒	☐	Clinical data
☒	☐	Dual use research of concern

Methods

n/a	Involved in the study	
☒	☐	ChIP-seq
☒	☐	Flow cytometry
☒	☐	MRI-based neuroimaging

Antibodies

Antibodies used	Immunocytochemistry: goat polyclonal anti-CD200R1 primary antibody (AF2554, 1:100; R&D); donkey anti-goat ALEXA 488 secondary antibody (A11055 488, 1:1000; Invitrogen).<br>Western blot: goat polyclonal anti-CD200R1 (sc-14392 M-21, 1:250; Santa Cruz Biotechnology Inc., Dallas, Texas USA); goat polyclonalanti-CD200 (AF3355, 1:500; R&D); mouse monoclonal anti-βtubulin (T4026, 1:50000; Sigma-Aldrich); horseradish peroxidase (HRP)-conjugated rabbit anti-goat (P0449, 1:2000; Dako) or goat anti-mouse (170-6516, 1:5000; Bio-Rad Laboratories, Inc.) secondary antibodies.
Validation	Immunocytochemistry: the use of decreasing concentrations of anti-CD200R1 antibody and the omission of the primary antibody were performed to stablish the specificity of the immunostaining and the optimal concentration to be used; we have been using this antibody for years to label CD200R1 in primary mouse microglial and mixed glial cultures and BV2 cells as well as in mouse CNS tissue (Dentesano et al., J Neuroinflammation 2012; Dentesano et al., Glia 2014; Valente et al., Front Cell Neurosci 2017).<br>Western blot: the presence of a unique clear band of the apropriate size was taken as indication of the specificity of the antibodies.

Human research participants

Policy information about studies involving human research participants

Population characteristics	Human monocyte and microglia-like cell cultures: nine healthy adults, age range 23-66 years; five females and four males.<br>Post-mortem human brain: eight control subjects (age range 56-90 years, four females and four males, post-mortem delay range: 4-20 h) and twenty-one Parkinson's disease patients (age range 50-92 years, five females and sixteen males, post-mortem delay range: 5-18 h).<br>Samples from iPSCs derived neurons: DNA samples from four control subjects (age range 47-66, two femlaes and two males) and 9 Parkinson's disease patients (age range 44-66, 5 females and 4 males).
Recruitment	Healthy adult volunteers were recruited as blood donnors for monocyte obtention.<br>Frozen post-mortem human brain samples from control subjects and Parkinson's disease patients were obtained from the tissue bank (Banc de Teixits Neurològics, Biobanc, Hospital Clínic de Barcelona, IDIBAPS, Barcelona, Spain).<br>DNA samples from iPSCs derived neurons were obtained in a previous study (Fernandez-Santiago et al., EMBO Mol Med 2015.
Ethics oversight	Ethics Commitee of the Hospital Clínic de Barcelona and Ethical Committees of the University of Barcelona and CSIC.

Note that full information on the approval of the study protocol must also be provided in the manuscript.
